# Why do patients decline surgical trials? Findings from a qualitative interview study embedded in the Cancer Research UK BOLERO trial (Bladder cancer: Open versus Lapararoscopic or RObotic cystectomy)

**DOI:** 10.1186/s13063-016-1173-z

**Published:** 2016-01-19

**Authors:** Emily Harrop, John Kelly, Gareth Griffiths, Angela Casbard, Annmarie Nelson, Published on behalf of the BOLERO Trial Management Group (TMG)

**Affiliations:** Marie Curie Palliative Care Research Centre, Cardiff University School of Medicine, Division of Population Medicine, 1st Floor Neuadd Meirionydd, Heath Park Way, Cardiff, CF14 4YS UK; Division of Surgery and Interventional Science, UCL Medical School, University College London, 74 Huntley Street, London, WC1E 6AU UK; Southampton Clinical Trials Unit, Faculty of Medicine, University of Southampton, Southampton, SO16 6YD UK; Wales Cancer Trials Unit, Cardiff University School of Medicine, 6th Floor Neuadd Meirionydd, Heath Park Way, Cardiff, CF14 4YS UK

**Keywords:** Qualitative, RCT, Bladder cancer, Surgery, Randomisation

## Abstract

**Background:**

Surgical trials have typically experienced recruitment difficulties when compared with other types of oncology trials. Qualitative studies have an important role to play in exploring reasons for low recruitment, although to date few such studies have been carried out that are embedded in surgical trials.

The BOLERO trial (Bladder cancer: Open versus Lapararoscopic or RObotic cystectomy) is a study to determine the feasibility of randomisation to open versus laparoscopic access/robotic cystectomy in patients with bladder cancer. We describe the results of a qualitative study embedded within the clinical trial that explored why patients decline randomisation.

**Methods:**

Ten semi-structured interviews with patients who declined randomisation to the clinical trial, and two interviews with recruiting research nurses were conducted. Data were analysed for key themes.

**Results:**

The majority of patients declined the trial because they had preferences for a particular treatment arm, and in usual practice could choose which surgical method they would be given. In most cases the robotic option was preferred. Patients described an intuitive ‘sense’ that favoured the new technology and had carried out their own inquiries, including Internet research and talking with previous patients and friends and family with medical backgrounds. Medical histories and lifestyle considerations also shaped these personalised choices. Of importance too, however, were the messages patients perceived from their clinical encounters. Whilst some patients felt their surgeon favoured the robotic option, others interpreted ‘indirect’ cues such as the ‘established’ reputation of the surgeon and surgical method and comments made during clinical assessments. Many patients expressed a wish for greater direction from their surgeon when making these decisions.

**Conclusion:**

For trials where the ‘new technology’ is available to patients, there will likely be difficulties with recruitment. Greater attention could be paid to how messages about treatment options and the trial are conveyed across the whole clinical setting. However, if it is too difficult to challenge such messages, then questions should be asked about whether genuine and convincing equipoise can be presented and perceived in such trials. This calls for consideration of whether alternative methods of generating evidence could be used when evaluating surgical techniques which are established and routinely available.

**Trial registration:**

Trial registration number: ISRCTN38528926 (11 December 2008).

## Background

Surgical trials have typically experienced recruitment difficulties compared with other types of oncology trials in the UK [1]. There are several reasons for this suggested in the medical literature. On the one hand, the culture amongst surgeons is considered to be less research oriented, and surgeons less experienced in conducting trials, compared with other clinical specialties engaged in cancer medicine [1]. There are also specific challenges with using randomised trials to compare types of surgery, such as the impracticalities of blinding, resistance to randomisation amongst surgical teams, difficulties attaining clinical equipoise, and the enhanced training that is necessary for new treatments [1]. Following this, clinicians may also need to accept that there is uncertainty or equipoise about treatments that are established in usual practice [2]. That said, there are examples of current recruitment ‘success stories’ in surgical oncology trials [3], and novel attempts to incorporate blinding into such trials [4], suggesting that such challenges are not necessarily insurmountable. Finding ways to improve the evidence base for surgical procedure is imperative [1, 3], as is the need to do so in ways which minimise ‘research waste’ [5–7]. Improving the efficiency of trial processes, including recruitment, is one such way of accomplishing this, although to date this remains a ‘largely evidence-free zone’ [8].

Qualitative research has an important role to play in investigating and addressing recruitment issues in trials. Studies investigating patient perceptions and experiences of surgical trials have helped illuminate why such trials experience difficulties recruiting [2, 9–13]. In the ProtecT, SPARE and QUEST trials (trials in prostate cancer, bladder cancer and breast reconstruction surgery) patient preferences for particular treatment options undermined recruitment into these trials [2, 10–13]. In the ProtecT and SPARE trials recruiters were found to unwittingly pass on their preferences to patients [10, 12], including the use of ‘loaded’ terminology [2, 11]. In the CLasP trial (which compared treatments for men with symptoms of benign prostatic disease) it was also observed that clinicians played a role in eliciting preferences and deciding who could participate [9].

Participants in these trials also experienced difficulties with the concept of randomisation. Whilst participants understood the principles of randomised design (i.e, chance, comparison and equipoise) [10], they struggled to accept randomisation because they could not accept ‘equipoise’; that the clinician was genuinely uncertain and the treatments similarly effective [2, 10, 11]. Participants in one trial also had expectations that their clinical appointments would be used to make individualised treatment decisions [9]. Such examples, therefore, suggest that it is not necessarily patient (mis)understandings of trial/medical information that is critical to patient decision-making, as is often assumed [11], but the culturally situated expectations that patients have of their ‘experts’; an argument which has been well-developed in qualitative research investigating public uptake of science [14–16]. This and other research also usefully highlights the different types of expertise, knowledge and forms of reasoning that people draw upon and develop when confronted with illness or perceived threats to their health and wellbeing [17–20], including ‘experience based expertise’ [17] and the culturally framed, personal, subjective and social knowledge, often described as ‘lay knowledge’ [18, 19].

The BOLERO trial (Bladder cancer: Open versus Laparoscopic or RObotic cystectomy) is a feasibility study that was developed on behalf of the National Cancer Research Institute (NCRI) Bladder Clinical Studies Group and funded by Cancer Research UK, coordinated by the Wales Cancer Trials Unit and Marie Curie Palliative Care Research Unit. The study aim was to determine the feasibility of randomisation to open versus minimal access (laparoscopic or robotic) cystectomy in patients with invasive bladder cancer, aged 18 or over. The secondary aims were to assess the safety and efficacy of minimal access cystectomy, and the reasons for non-acceptance of randomisation [21]. Open and minimal access surgery were routinely available to all patients in our six participating trial sites, regardless of whether or not they entered the study. Some of these centres were also offering robotic surgery to their patients (trial and non-trial); at the other centres laparoscopic was the only minimal access option available.

The trial was designed with a quantitative primary end-point of feasibility of randomisation, defined as >60 % of patients accepting randomisation, which would be used to determine whether a future randomised phase III trial, requiring many hundreds of patients, was possible to achieve. Within this trial an embedded qualitative study was included to explore factors relating to a decision to decline randomisation and patient experiences of trial recruitment processes. Although not designed to identify and implement changes to the feasibility trial, as in other studies [2, 10–13], it was intended that the findings could be used to make trial specific recommendations for improving recruitment practices and documentation should the trial have progressed to a full trial and more broadly be used to inform the design and conduct of similar trials in the future by contributing to the small amount of evidence in this area. A more detailed description of trial design is available on-line [21] and the quantitative outcome, including activity and safety of the treatment arms, will be reported elsewhere. For the background purposes of this paper, however, it is reported that the threshold of >60 % of patients accepting randomisation was not reached, and a full phase III trial would not, therefore, be considered feasible. This paper reports the qualitative outcome which not only enhances understandings of recruitment issues relating to this particular trial, but more widely, adds to the qualitative evidence described above, and identifies implications for the evaluation of similar surgical/technology interventions in the future.

## Methods

The aim of the qualitative study was to explore factors relating to a decision not to consent to randomisation between open surgery and minimal access surgery (robotic or laparoscopic), and participants’ experiences of recruitment processes in the BOLERO trial. The embedded qualitative design involved semi-structured interviews with up to 20 patients and an in-depth thematic approach to analysis of the data. This research was informed by a phenomenological perspective and interest in exploring the lived experiences of participants and their decision- making in the context of their life experiences, values and beliefs. For the purposes of this paper and our clinical audience, however, our results are presented as group themes which cut across patients’ views and experiences, but where relevant also identify the salient life context and stories of individual participants in relation to the theme being discussed.

Ethical approval for the BOLERO trial (including the qualitative study) was granted by South East Wales Research Ethics Committee in May 2010, and the trial was sponsored by Cardiff University. Ten participants were recruited into the qualitative study from three (out of a potential six) sites which were recruiting into the main BOLERO trial. Robotic surgery was not available at one of these three sites. The interviews were conducted between October 2011 and December 2012, and included eight male and two female participants, with an age range of 44 to 74, and a mean age of 63.5 years. Participants (P)2 and P10 were the only patients from the site without robotic surgery; all other participants had the option of receiving robotic surgery.

Informed consent was obtained for all participants. All patients who declined randomisation into the main BOLERO trial were given information about the qualitative study by their research nurse. Patients who wanted to participate in the qualitative study were consented into the study by their research nurse before being contacted by the qualitative researcher to make interview arrangements. When the minimum number of participants was deemed sufficient for meaningful analysis, and theoretical saturation was reached, recruitment was closed to the qualitative study. Just under a quarter of the patients who were eligible for this qualitative study participated in an interview.

In response to initial slow recruitment to the qualitative study, the study protocol was also amended to include a further study involving semi-structured interviews with the six research nurses recruiting to the BOLERO trial. Research nurses have immediate experience of screening, providing trial information to, and consenting prospective participants. In this trial, the research nurses would talk through the trial information in detail with patients, after the surgeon or another member of the clinical team had introduced and explained the trial to the patient during their clinical consultation. They were typically also the first point of contact for patients once they had made a decision on whether or not to enter the trial. It was thus considered that they would have good experience-based knowledge of why patients declined or accepted randomisation to the trial, as well as views on the process of presenting and explaining the trial information, and any difficulties that this may present. Semi-structured interviews with the research nurses involved in this trial were used to explore their views and experiences with regards to recruitment and to provide insights into any problems and issues specific to this aspect of the trial.

Ten semi-structured interviews were carried out with patients in a location of the participants’ choice; patient’s homes (*n* = 4); their place of work (*n* = 1) and by telephone (*n* = 5). Interviews were conducted by a female researcher with experience in cancer and healthcare research, but no clinical background. Interviews lasted between 20 and 45 minutes and explored patients’ experiences of trial information and recruitment processes, reasons for declining randomisation into the main trial and attitudes towards medical research (interview topics summarised in Table 1).

**Table 1:** Summary of interview topics

Topics for patient interviews	Topics for nurse interviews
Trial information and recruitment processes • Experience of being told about the trial • Understandings of trial purpose and principles, e.g. randomisation • Views on participant information sheet • Other sources of information accessed about types of surgery/trial • Prior knowledge of and preferences for surgical methods Decision-making • Reasons for declining randomisation • Choice of surgery and reasons given • Influence of clinical team and others on decision-making • Reflection on choices made and decision-making process Experience of and attitudes towards medical research • Views on randomisaton as process of allocating treatment • Participation in previous and (potential) future research studies	Recruitment practices • Professional background and time on trial • Own and others’ duties and responsibilities with regard to the trial • Description and reflection on information giving process for trial/other options available to patients Patient responses • Recall of patient responses to trial information • Views on patients’ reasons for declining randomisation • Views on patients’ reasons for accepting randomisation • Reflection on differences between patients who accept/decline trial General reflections • Compare and contrast with recruitment experiences of other trials • Key challenges of this trial and suggestions for future trial design and conduct

Interviews were audio-recorded, transcribed verbatim and uploaded to NVivo 9 software. Data analysis was undertaken by two researchers who shared the analysis of transcripts, following an in-depth thematic approach [22]. Group results were analysed for consistent themes using techniques of coding and comparison. A coding framework for emergent themes was developed by the researcher. This was an iterative process, moving between the data and the analytical concepts to develop codes and concepts grounded in the data. Higher level abstractions of codes were decided and results were verified by the research team by independent review of a selection of transcripts.

Unfortunately, only two of the six research nurses agreed to take part in an interview and one of these interviews failed to record; the data from this interview are from notes taken during the interview and written up by the researcher from memory immediately afterwards. This seriously limits the contribution of these interviews to the study results. However, although this data cannot be interpreted as ‘findings’ in their own right, they do provide some useful context for the findings of the patient interview study and are reported as such.

## Results

### Understandings of and attitudes towards trials

In general, these study participants were well-informed and had a good understanding and appreciation of the trial and medical research in general. Participants were satisfied with the study information which they were given and felt comfortable asking questions, although some also described feeling overloaded by the amount of clinical and trial information which they received at this time and needed to consider in their decision-making:

I did fully understand um enough um to be going on with. What I did feel that I was overloaded with information from everything it was another lot of information I had to take on board, if you see what I mean because there were so many other things I had to think about, about what sort of bladder I was going to have, whether it was going to work you know, all this sort of thing going on as well um so this was just yet another thing I had to sort of um you know think about. (P9)

Most participants recognised the value of research for generating evidence to improve treatments for future generations, and several patients had previously participated in other medical research studies. Most participants seemed to correctly understand the purpose of the BOLERO trial as being to compare the different types of surgery, and felt happy with their level of understanding. Participants understood the basic concept of randomisation as having treatment allocation left to chance. Some patients made sense of randomisation as a ‘50-50 split’, while others stressed the role of the computer, and made use of metaphors of ‘pot luck’ and ‘letting the dice decide’ to make sense of the process:

When (research nurse) said it was a 50/50 split, you know random split and I might actually, having had all these tests and think how good this procedure was, I might actually be put back into a 50 % of having the more invasive surgery. (P6)

### Choosing keyhole and robotic surgery

The main reason for patients declining the trial was because they had developed a preference for the type of surgical method they wanted and in usual practice could choose which method they would receive. With the exception of one patient who opted for open surgery, all other patients chose keyhole and where it was available opted for robotic surgery. However, one of these patients ultimately rejected surgery altogether, choosing to receive bacille Calmette-Guérin (BCG) therapy instead (a vaccine given directly into the bladder). These patients were thus unwilling to relinquish control by accepting randomisation and with it the possibility that they might not receive their preferred option:

If you didn’t know about it as you know if, if, if you surgical team were randomising what they did and I would never know then that would have been an irrelevance to me but once I knew there was a choice. I wanted to exercise that choice. (P3)

In line with the perspectives of patients recruited from the same centre, one of the nurses described strong patient preferences for robotic surgery, which they considered to be the key factor undermining trial recruitment. This was attributed to the fact that the centre was already offering robotic surgery to patients, which made it difficult to convey messages of equipoise and uncertainty when discussing the trial:

I think because they’re offered the robot initially (.) although they are told you know that normally here it is robotic surgery um that sort of (.) sort of seals the deal if you will … the patients think well the robot’s here I might as well have that. (RN2)

Both nurses considered that many patients felt uncomfortable leaving surgery decisions to chance when the alternative was to make an informed choice and could opt for their preferred treatment with guidance from their clinical team:

The main reason is that they know they can have the robot here anyway and some don’t like the fact that it’s being chosen for them you know that it’s basically a computer saying right this is what will happen. (RN2)

Patient preferences for type of surgery were linked to their understandings of the different risks and benefits attached to each method. Most patients saw the benefits of keyhole/robotic to be that it was less invasive, more precise, and requiring smaller cuts. This was understood to mean that there would be less scarring, smaller risk of infection and a shorter recovery period than for open surgery. The only patient who preferred open surgery, by contrast, considered that he would prefer to have one big scar as opposed to multiple small scars:

I wanted the best option and the least invasive option and that seemed to be the smallest cut and the better surgery seemed to be the robot. (P1)

### Intuition and common sense

For several patients, their preference for robotic surgery seemed intuitive; it made sense to them that this procedure would be the better one. This was because they understood it to be ‘more delicate and more precise’, which should in turn facilitate a quicker recovery. Some also valued the fact that it was a new, technologically advanced procedure and felt that they would be taking part in something pioneering:

This wonderful new robotic arm that’s gonna help me heal faster by having this procedure so that I can get on with a normal life … I’ll be able to recover faster and be back at work and … won’t be a normal life but a more normal life … the procedure would be more compatible with my body and recovery and stuff. (P8)

Conversely, and illustrative of patients’ idiosyncratic preferences, the only patient to express a preference for open surgery expressed misgivings about being operated on by a robot:

Uh I just didn’t think it was right … I’d rather have a person doing it, and a person saying to me you know this is what we’re doing. (P2)

### Patient research and enquiry

Although for some patients their preferences seemed more intuitive, many had also carried out their own inquiries which included research using the Internet, talking with previous patients and consulting with friends and family with medical backgrounds. The research that these patients carried out themselves clearly influenced their preferences for robotic surgery. This first patient described how a perceived lack of direction in his initial consultation led him to carry out his own research, which in turn seemed to pre-empt the discussions and decision-making which took place in his subsequent appointments. He had entered these consultations with a clear idea of the treatment which he wanted, and as a result could not recall hearing much about the trial at all:

I have uh done my own little research and in response to ‘well, uh what do you think?’ I said that I was interested in the neo-bladder operation, in particular the robotics … I read about (a patient who had this particular operation) and then I looked into it a little bit more and so the next time, I, I said, I expressed interest in the robotic one, that was there. (P5)

Other patients used Internet-based sources to supplement the ‘official’ information that they were given in the hospital. This information seemed to reinforce rather than instigate their treatment preferences and decision-making. Several patients, sometimes on the recommendations of their surgeons had also watched online videos of the robot, while others had met with former patients who had received the surgery, an experience which they found reassuring. While all patients were clear that their decisions were their own, many described how they received information and advice from family members and friends. One half of patients described input from friends or family members with medical backgrounds or connections, which they used to help them make informed choices:

My sister lives in America and she, her in-laws work in hospitals, micro-biologists, technicians sort of thing. So I got them to um tell me what they thought, they’d all worked in where the robot had been um and came back with you know way to go you know, if you get a choice don’t do anything else sort of thing. (P9)

### Individual circumstances and characteristics

Patients took into account their own medical histories and ways of life when considering the different options available to them as they made personalised treatment choices. Some described illness histories, health events and concerns over their current fitness in relation to their preferences for the least invasive options:

When they said about surgery I thought ‘oh cuts’ and that and healing. I knew that I had a dog bite years ago and that took weeks to heal (and) I thought ‘God, if I’m going to have a cut here and it’s going to heal, it’s going to be agony for weeks sort of thing so as soon as they said about that I said no, I wasn’t interested’ (laughs). (P4)

Another patient described how her past experiences of cancer intensified her determination to take up treatment and return to normal family life as rapidly as possible. Similarly, participant one (below) described how his 20-year battle with bladder cancer meant that he felt a heightened need for control and decisive action as opposed to leaving anything to chance by entering the trial:

I think I’ve had 22 years of bladder cancer, most of its been superficial and I’ve had, I imagine I’ve had at least ten periods of different types of chemotherapy … I’ve had BCG a number of times and it used to disable me, it was so bad, like … and so I felt like I didn’t want any more nonsense. I just wanted to get on with it and get the bladder out. (P1)

Some patients also described how their particular lifestyles and life concerns favoured certain treatment options, for example returning to work to meet particular deadlines or keeping up with ‘energetic’ leisure pursuits following keyhole surgery. This was most extreme for the patient in his mid-40s who, at the point of interview, had decided against surgery altogether (opting instead for BCG), due in large part to the impact that the operation would have on his sex life, which was of particular concern to him given his young age and lifestyle:

My only concern is being a lot younger than the gentleman that had it done (..) vanity as it is I still quite enjoy a sex life. This gentleman was like in his 60s … well if I don’t have to have surgery why do it even if there’s the slimmest of chances, it’s got to be worth a go. (P8)

### Clinical influences

Participants were influenced by their clinical team. This influence occurred through direct and indirect messages which patients interpreted from their clinical encounters. Only a small number of patients felt that their consultants conveyed equipoise or messages of uncertainty with regards to the different surgery options. These surgeons would not explicitly state which would be the better method with a justification of ‘not being sure yet’:

I think he was trying to be very much on the fence … he said that I would be accepted for robotic surgery, (.)… I didn’t really get (..) a feeling from him one way or the other. (P9)

Others recalled the more general positive messages about keyhole and robotics which they received from their clinicians, which convinced them that this was the route to go down:

Well, he was saying about you know smaller cuts and that sort of thing and that uh, less to heal and quicker, it’ll heal quicker and all that sort of thing yes, everything in favour of it as I say … the fact that that particular surgeon said ‘well at least it’s got a steadier hand than me’; I thought well that’s something. (P4)

Many patients also interpreted more indirect or subliminal messages during their clinical encounters. A couple of patients recalled the positive discussions which they had with their anaesthetists during their pre-operative assessments, which in delivering good news about their fitness to have keyhole surgery, helped to reinforce impressions of this being the better method:

When I went to my pre-op assessment with the anaesthetic team, which involved riding a bike mainly and having my heart tested and things, they said to me oh you seem fine, your hearts good, your blood pressure blah de blah is all good um so you should be fine for the procedure and my overall impression was (.) that if you were fit and well and good enough (.) physically to do it, it was the best choice … that everyone seemed to think that it was a really good thing that I was fit and well because I could have this procedure. (P6)

A number of patients were also aware of the strong reputations of their surgeons in delivering pioneering robotic surgery, and from this deduced that the robotic option would also most likely be the surgeon’s preferred method:

He’s quite well-renowned for robotic stuff so I imagine that he would have gone for robot … I don’t know, I mean I would have thought he would because that is what they are doing. They are going for robot surgery and hopefully it’s going to be much better. (P1)

Patients not only described confidence in the surgeon when it came to the robotic operation, they had also felt that the method was well established and ‘tried and tested’:

There seems to be a procedure that they’ve tried and tested over the last 3 years. Well it says here 600 people have had the procedure just in (location name) … So I don’t feel like it’s um a guinea pig situation. So um, um so I’m taking it at face value really that that’s the one they think I should have. (P6)

Similarly, whilst this next patient described how his surgeon had countered his efforts to obtain ‘expert’ advice from him by suggesting a lack of definitive evidence, the length of time in which the operation had been practiced was enough to reassure them:

I said to him ‘you’re the expert, you should be advising me’. But he said ‘oh but we’re not sure yet’ um ‘what’s the best’ and I said ‘well how long have you been doing for?’ And I think he said ‘2 years’ but I could be wrong. And so I said ‘I’ll go for the robot’ because you know it might be less invasive and easier to get better’. (P1)

Both nurses felt that they presented equipoise as best they could by emphasising that it is not known which method is best. However, they also reflected on how this could be difficult because many patients had already formed clear ideas about which treatment they wanted:

The main message …with the trial is, because we don’t know which one is the better procedure in outcomes after surgery, i.e. um recovery times,(.) infection rates, pain control all those sorts of things. But it’s you know the sorts of things we say to the patients, because obviously they tend to latch onto one arm of the trial and think that is the best one. It’s quite difficult to sort of get them back on an even keel to think well you know we don’t know whether that is or not. (RN2)

It was felt that the consultants were effective at explaining the different treatment options to patients, but also considered that some consultants may pass their own preferences for type of surgery onto their patients:

Some of them do (..) um I think the consultant here likes his robotic surgery but he also likes the trials as well so that’s why he (.) you know he explains everything so well to the patients I think so they are fully informed when they do go away with the information sheet. (RN2)

When asked for their views on why some patients accepted randomisation, one of the nurses considered that having an existing relationship with the trial staff seemed to be the key factor due to feelings of trust and familiarity:

I think mainly because some of the patients that we randomise into the trial here were patients that I’d treated before in another trial, so I think sometimes that has a lot to do with it, that they know the people that are in the room … I’d already looked after them for 6/7 months beforehand, given them treatments, been at the clinic appointments and I think they’re a little bit (.) more trusting, I don’t know if trusting is the right word, but I think because they feel comfortable in the room with the people that are there. (RN2)

The other research nurse considered that the only noticeable difference between consenters and non-consenters was the emotional wellbeing of the patient, with more distressed patients less likely to enter the trial. Although only based on two perspectives, these insights again indicate the relevance of patients’ emotions, the clinic environment and the relationships that patients have with their clinicians for decision-making regarding trials.

### Preferences for greater clinician input or involvement

Many patients would have liked greater direction from their surgeon when making these decisions. Although patients ultimately formed preferences for the type of surgery they would receive, some patients also described how their primary preference was for guidance and direction from their surgeon when making these choices. In other words, it was not that they had a particular preference for a type of surgery at the outset; rather that they wanted the decision to be reached with input from their clinician, and experienced a sense of unease and frustration when this was not accomplished:

I said the only aspect of the trial I wasn’t happy with was the randomisation because I felt that the decision should be arrived at as a sort of a clinician decision between the consultant and the patient. (P3)

I mean the fact of the matter is I don’t know anything and so asking me that is just uh, just not on … I would like to have a narrative account of what the problem, of how it’s going be and what it’s, what the alternatives are and I never did get it *…* So I went and made up my mind on my own uh which now that I’m over it I find that’s OK. I’m not regretting it (.) but um it filled me with unease. (P5)

Both of these (older) patients also reflected on how current models of patient-led consultation were ‘at odds’ with the values, expectations and preferences of their generation for authoritative guidance from their physicians:

Well, I, I come from an age where you do as your doctor tells you whereas someone half my age (.) might arrive at a consultation with a ream of print outs from his PC explaining (what) he wanted done. Um I don’t (.) think that’s the right way forward anyway. (P3)

## Discussion

This paper contributes to the small amount of evidence from embedded qualitative studies which help to explain recruitment difficulties experienced by surgical trials. It identifies a perceived lack of equipoise between trial arms amongst our ‘non-consenter’ participants and draws attention to a number of factors which explain patient preferences and choices. As such, this study adds new insight to previous similar findings in this area and identifies important implications for future research aimed at evaluating the effectiveness of surgical/technology innovations, particularly those already available as part of routine practice.

In general, these study participants were well-informed and had a good understanding and appreciation of the trial and medical research more broadly. Most participants seemed to correctly understand the purpose of the trial as being to compare the different types of surgery, and felt happy with their level of understanding. As in previous surgical trials, participants understood the basic concept of randomisation as having treatment allocation left to chance, but could not accept this method of treatment allocation because they could not accept the supposed ‘equipoise’ in this trial and that their clinician was genuinely uncertain [2, 10, 12].

As in previous studies, the main barrier to recruitment into this surgical trial seems to have been the emergence of clear treatment preferences amongst patients [2, 9–13]. In this embedded study, most of the patients who declined randomisation did so because they had preferences for the surgical method they would be given, which in most cases was the robotic option. The ability of these patients to choose their preferred treatment option in usual care meant that they were unwilling to relinquish control by accepting randomisation and with it the possibility that they might not receive their preferred option, as similarly demonstrated in a feasibility trial of childhood strabismus surgery [23]. Patient preferences for type of surgery were linked to their understandings of the different risks and benefits attached to each method. Most patients saw the benefits of keyhole/robotic surgery to be that it was less invasive, requiring smaller cuts and producing less scarring, smaller risk of infection and a shorter recovery period than for open surgery.

Patients drew on multiple forms of knowledge and expertise to inform their decision-making. Many had sought additional information from ‘accredited’ sources of expertise, such as published research studies available on the Internet, and friends and family members with medical backgrounds, whilst a few looked to the narrative accounts and ‘experience-based’ expertise of patients who had already undergone surgery [17]. Patients also drew on their own personal, subjective and culturally framed knowledge [18, 19]. For several patients their preference for robotic surgery seemed intuitive. It made sense to them (within their cultural frames of reference) that this would be the better option; it was new and technologically advanced whilst also perceived as sufficiently ‘tried and tested’. Patients also took into account their own illness histories and lifestyles when weighing up the different options available to them, to make what could be considered personalised treatment choices. These findings highlight the context dependent nature of knowledge and decision-making [24, 25], and illustrate well the argument that patients judge treatment outcomes subjectively based on their personal circumstances [25]. This causes difficulties for the practice of clinical equipoise in trials in so far as different people will attach different meanings to presented outcomes and may expect that these are taken into consideration when planning treatment pathways.

Participants had also been influenced by their clinical team, from whom they had expected and sought expert guidance and direction. This influence occurred through direct and indirect messages. Only a small number of patients felt that their consultants conveyed equipoise or messages of uncertainty with regards to the different surgery options. As in previous surgical trials many patients felt that their surgeon had openly favoured a particular trial arm [2, 10–12], in this case the robotic method. Many patients also interpreted indirect or subliminal messages during their clinical encounters. These ‘indirect’ cues included positive comments made about fitness and suitability for robotic surgery during pre-operative assessments; the strong reputations of their surgeons in delivering pioneering robotic surgery (and assumed preferences for this technique), and the fact that the ‘new method’ had become well- established at these centres. In some ways this contrasts with the findings of an embedded study in another surgical trial, in which participants responded to loaded terminology in favour of the established (as opposed to ‘experimental’) treatment [2]. What both studies more importantly illustrate, however, is the salience of hidden messages in unwittingly shaping patient preferences, and the openness of patients to such cues as they actively seek out ‘expert’ influence and confirmation.

Following this, a number of patients were explicit about wanting greater input from their consultants when making their treatment decisions [23]. This preference aligns with research on decision-making preferences in healthcare, which notes that whilst patients welcome the opportunity to participate in decision-making it appears they do not necessarily want to be responsible for decisions, with a sizeable minority to a half preferring passive roles [26–28], compared with a much smaller (and generally better educated, younger) minority who prefer purely autonomous roles [27–29]. In this study, it was the older (but well-educated) patients who expressed greatest frustration at the lack of direction, even reflecting on what could be conceptualised as a ‘culture clash’ between the values and expectations of their generation and current medical models of patient-led choice and calculated risk assessment [14–16]. These patients struggled to accept randomisation not just because of the preferences which they formed but because they had an underlying expectation that their clinicians would make treatment decisions based on individual clinical assessments [9], thus further highlighting the need to contextualise knowledge, risk and decision-making [14, 16, 24].

### Limitations and implications for further research

Recruitment to the qualitative study was slow and just ten patient interviews were carried out. Although these patients represented a homogenous group and sufficient data were gained from these interviews to reach saturation and enable meaningful analysis, it should also be noted that a larger proportion of non-consenters to the clinical trial declined to take part in the qualitative interview. It is, therefore, possible that our sample included participants who were more positive towards medical research than the wider population of non-consenters who were eligible for this embedded study. This in turn may have biased our results in other ways given that a key concern of the study was with patient perceptions of, and engagement with research. However, it is unlikely that this bias should detract significantly from our main findings on patient preference and availability of choice as barriers to trial recruitment.

Whilst our interviews with research nurses were informative and provided some useful context it was disappointing that only two out of six nurses agreed to be interviewed, and only one of these interviews was recorded, thus limiting any conclusions that can be drawn from this data. Interviewing patients who decline the trial is only one part of the picture, a more comprehensive investigation of recruitment issues could have included observations of recruitment settings and practices, analysis and patient assessments of study documents and consultations and interviews with a larger sample of clinician recruiters, as well as participants who enter the trial. Ideally, this type of in-depth qualitative investigation of trial processes should take place before, or in the early stages of, the feasibility trial with formal mechanisms for feeding back key findings and designing and implementing practical changes to recruitment materials (e.g. DVDs, communication checklists) and staff training which could help to achieve a more effective presentation of equipoise. Such approaches have been shown to improve recruitment into trials [10, 12], and are recommended for further studies of this kind. Given the apparent recruitment ‘success’ of a similar trial being conducted in the US [3], exploration of inter-continental differences in trial recruitment practices could also be worthwhile.

## Conclusion

Patients like to exercise informed choice over the type of surgery they receive, and they like this choice to be informed by their clinical team. In contrast with many cancer ‘drug trials’, where trial entry might be the only chance of receiving the ‘experimental’ treatment, for surgical trials where the ‘new technology’ is routinely available to patients there will likely be difficulties with recruitment. Going forward, an alternative option for similar technology trials could be to use centres which do not yet routinely use the technology under investigation, with access to the ‘new’ procedure restricted to trial patients at these sites, thus incentivising patient entry into the trial.

Greater attention could also be paid to how messages about treatment options and the trial are conveyed, including the more subliminal messages that pervade the whole clinical setting; a strategy which was tried and found to be effective in improving recruitment for another surgical trial [10, 12]. However, if it is too difficult to challenge such messages, for example if the ‘experimental’ treatment is already entrenched in culture and practice (as seemed to be the case in this trial), then questions should be asked about whether equipoise can be presented and perceived with conviction in such trials. Moreover, accommodating patient wishes for clinician input and personalised consideration of individual circumstances and characteristics, as is expected by patients and practised in other areas of medical care, presents a challenge in clinical trials and can leave patients feeling frustrated and anxious if they perceive that this is not being accomplished in their consultations. Clinical equipoise is a difficult concept for patients to accept and feelings of anxiety and frustration are probably more likely to occur where there is not genuine conviction and belief in clinical uncertainty amongst recruiting clinicians.

These issues challenge the feasibility of similar trials in the future [11], and have implications for current agendas on minimising ‘trial waste’; absent in which have been questions concerning the appropriateness of using randomised control trial (RCT) designs when evaluating certain types of interventions [5–8]. These findings call for consideration of whether alternative trial designs or methods of generating evidence could be used when evaluating surgical techniques which have already become established and routinely available, and where there are likely to be clear patient and physician preferences [1, 11, 13, 30].

## Abbreviations

BCG: bacille Calmette-Guérin
CRUK: Cancer Research UK
NCRI: National Cancer Research Institute
P: participant
RCT: randomised control trial
RN: research nurse
TMG: Trial Management Group

## References

[1] National Cancer Research Institute (NCRI) (2012). Challenges and opportunities in surgical cancer research in the UK.

[2] Paramasivan S, Huddart R, Hall E, Lewis R, Birtle A, Donovan J (2011). Key issues in recruitment to randomised controlled trials with very different interventions: a qualitative investigation of recruitment to the SPARE trial (CRUK/07/011). Trials.

[3] Smith N, Castle P, Gonzalgo M, Svatek R, Weizer A, Montgomery J (2015). The Razor (randomized open vs robotic cystectomy) trial: study design and update. BJU Int.

[4] Avery K, Metcalfe C, Berrisford R, Paul Barham C, Donovan J, Elliott J (2014). The feasibility of a randomized controlled trial of esophagectomy for esophageal cancer − the ROMIO (Randomized Oesophagectomy: Minimally Invasive or Open) study: protocol for a randomized controlled trial. Trials.

[5] Chalmers I, Glasziou P (2009). Avoidable waste in the production and reporting of research evidence. Lancet.

[6] Macleod M, Michie S, Roberts I, Dirnagl U, Chalmers I, Ioannidis J (2014). Biomedical Research: increasing value, reducing waste. Lancet.

[7] The Lancet. http://www.thelancet.com/series/research. Accessed 5 April 2015

[8] Trial Forge. www.trialforge.org. Accessed 5 April 2015

[9] Featherstone K, Donovan J (2002). ‘Why don’t they just tell me straight, why allocate it?’ The struggle to make sense of participating in a randomised controlled trial. Soc Sci Med.

[10] Mills N, Donovan J, Smith M, Jacoby A, Neal D, Hamdy F (2003). Perceptions of equipoise are crucial to trial participation: a qualitative study of men in the ProtecT study. Control Clin Trials.

[11] Moynihan C, Lewis R, Hall E, Jones E, Birtle A, Huddart R (2012). The Patient Deficit Model Overturned: a qualitative study of patients’ perceptions of invitation to participate in a randomized controlled trial comparing selective bladder preservation against surgery in muscle invasive bladder cancer (SPARE, CRUK/07/011). Trials.

[12] Donovan JL, Mills N, Smith M, Brindle L, Jacoby A, Peters TJ (2002). Improving the design and conduct of randomised trials by embedding them in qualitative research: the ProtecT study. BMJ.

[13] Winters Z, Emson M, Griffin C, Mills J, Hopwood P, Bidad N (2015). Learning from the QUEST multicentre feasibility randomization trials in breast reconstruction after mastectomy. Br J Surg.

[14] Wynne B, Irwin A, Wynne B (1996). Misunderstood misunderstandings: social identities and the public uptake of science. Misunderstanding science? The public construction of science and technology.

[15] Lash S, Lash S (1996). Introduction. Risk, environment and modernity: towards a new ecology.

[16] Irwin A, Michael M (2003). Science, social theory and public knowledge.

[17] Collins H, Evans R (2002). The third wave of science studies: studies of expertise and experience. Soc Stud Sci.

[18] Williams GH, Popay J, Kelleher D, Gabe J, Williams GH (2006). Lay knowledge and the privilege of experience, 2nd edition. Challenging medicine.

[19] Potts L (2004). An epidemiology of women’s lives: the environmental risk of breast cancer. Crit Publ Health.

[20] Elliott E, Harrop E, Williams GH, Bennett P, Calman S, Curtis S, Fischbacher-Smith D (2010). Contesting the science: public health knowledge and action in controversial land-use developments. Risk communication and public health.

[21] ISRCTN registry. http://www.controlled-trials.com/ISRCTN38528926. Accessed 16 November 2015

[22] Braun V, Clarke V (2006). Using thematic analysis in psychology. Qual Res Psychol.

[23] Buck D, Hogan V, Powell CJ, Sloper JJ, Speed C, Taylor RH (2015). Surrendering control, or nothing to lose: parents’ preferences about participation in a randomised trial of childhood strabismus surgery. Clin Trials.

[24] Wynne B (1991). Knowledge in context. Sci Technol Hum Values.

[25] Lilford R (2003). Ethics of clinical trials from a bayesian and decision analytic perspective: whose equipoise is it anyway?. Br Med J.

[26] Cox K, Avis M (1996). Ethical and practical problems of early anti-cancer drug trials: a review of the literature. Eur J Cancer Care.

[27] Deber R, Kraetschmer N, Urowitz S, Sharpe N (2007). Do people want to be autonomous patients? Preferred roles in treatment decision-making in several patient populations. Health Expect.

[28] Levinson W, Kao A, Kuby A, Thisted R (2005). Not all patients want to participate in decision making. A national study of public preferences. J Gen Intern Med.

[29] Rothenbacher D, Lutz M, Porzolt F (1997). Treatment decisions in palliative cancer care: patients’ preferences for involvement and doctors’ knowledge about it. Eur J Cancer.

[30] McCulloch P, Taylor I, Sasako M, Lovett B, Griffin D (2002). Randomised trials in surgery: problems and possible solutions. BMJ.

